# Workplace violence among nurses working in public hospitals in Northern Ethiopia; a mixed method study

**DOI:** 10.3389/fpubh.2025.1568264

**Published:** 2025-06-26

**Authors:** Mamush Gidey Abirha, Kibrom Berhanu Gebreslassie, Gerezgiher Buruh Abera, Binyam Gebrehiwet Tesfay, Fissha Brhane Mesele, Fiseha Abadi Gebreanenia, Kelali Goitom Weldu, Willi Bahre, Guesh Teklu Woldemariam

**Affiliations:** ^1^Department of Nursing, College of Medicine and Health Sciences, Adigrat University, Adigrat, Ethiopia; ^2^School of Nursing, College of Health Sciences, Mekelle University, Mekelle, Ethiopia; ^3^Department of Nursing, College of Medicine and Health Sciences, Raya University, Maichew, Ethiopia; ^4^Department of Nursing, College of Health Sciences, Axum University, Axum, Ethiopia; ^5^Tigray Regional Health Bureau, Mekelle, Ethiopia

**Keywords:** workplace violence, nurses, Ethiopia, mixed study, public hospitals

## Abstract

**Background:**

Workplace violence has become a warning universal phenomenon, particularly affecting healthcare workers, especially nurses. This study aimed to assess the prevalence of workplace violence and its associated factors among nurses working in public hospitals in Northern Ethiopia.

**Methods:**

A mixed-methods study was conducted at a hospital in Northern Ethiopia among 416 nurses using a self-administered questionnaire. A simple random sampling technique was employed to select the participants. Descriptive, bivariate, and multivariable logistic regression analyses were performed using the Statistical Package for Social Sciences (SPSS) version 27. For the qualitative part, critical case purposive sampling was used to select respondents, and data were collected through face-to-face, in-depth interviews. Finally, inductive thematic analysis was performed on the data using ATLAS.ti 23.

**Result:**

The prevalence of workplace violence among nurses in the last 12 months was 62.8% (95% CI, 57.5–76.3). Working in the emergency department was associated with a significantly increased risk (AOR = 4.97, 95% CI: 1.10–22.70, *p* = 0.039). Conversely, being married (AOR = 0.58, 95% CI: 0.34–0.98, *p* = 0.046) and having a good work performance (AOR = 0.59, 95% CI: 0.36–0.96, *p* = 0.03) were associated with a significantly decreased risk. Additionally, having 1–5 staff members in the same working area (AOR = 3.40, 95% CI: 1.12–10.40, *p* = 0.030) and working alone (AOR = 2.34, 95% CI: 1.34–4.10, *p* = 0.003) were significant factors. Nurses perceived understaffing, shortage of drugs and supplies, lack of security, and lack of management attention to workplace violence as the main reasons behind these incidents.

**Conclusion:**

Workplace violence among nurses was found to be significantly high in the study area. We recommend that it is essential for the health sector to establish a strong system for preventing and reporting incidents of workplace violence by involving medical personnel.

## Introduction

Workplace violence is any act or threat of physical violence, harassment, intimidation, or other threatening disruptive behavior that occurs at the work site. It ranges from threats and verbal abuse to physical assaults and even homicide (1).

Workplace violence (WPV) has sadly become a widespread and concerning phenomenon around the world, posing a serious threat to healthcare workers, particularly nurses. Despite the lack of complete data on its full extent, studies conducted across various countries, in both high-income and low-income and the result consistently reveal a disturbing trend. Approximately 25% of all workplace violence incidents occur within the healthcare sector. And nurses, particularly, face a high risk of abuse, making this a significant and endemic problem within the healthcare field (2, 3).

Health workers are 16 times more likely to experience WPV, and among healthcare workers (HCWs) nurses are three times more likely to experience violence at work. In an International Council of Nurses study (ICN), 1 in 4 nurses reported experiencing physical violence at work in the previous year (4, 5).

Due to their frontline role, nurses are more vulnerable to violence than other hospital staff. This increased risk stems from their direct interactions with patients and their relatives. This troubling reality poses a significant hurdle, with over half of nurses globally experiencing at least one violent incident within the past year (6). In China alone, an alarming 62.7% of nurses reported facing workplace violence in the last 12 months (7). Similarly, a staggering 67% of nurses in Saudi Arabia reported experiencing workplace violence (8).

Workplace violence against nurses in Africa is alarmingly prevalent, with varying degrees across different countries. Reports reveal concerning figures: 65.7% in Egypt, 86.1% in South Africa, 53.5–65% in Nigeria, and 73.9% in Ghana (9).

Workplace violence in healthcare harms workers physically, mentally, and financially, impacting patient care and the healthcare system (8, 10). Despite international efforts, workplace violence in healthcare remains a major problem (11). So far there is no significant change in minimizing the incidence of WPV.

Nurses in war-torn Mekelle, Northern Ethiopia face high rates of workplace violence, with limited data on its prevalence status and forms. Northern Ethiopia has experienced conflict and instability in recent years, which may exacerbate stress and aggression in healthcare settings. In addition, cultural, political, and organizational dynamics in the region differ from those in other parts of the country and may influence the type and frequency of workplace violence. There is also a general lack of comprehensive, region-specific data on workplace violence in Northern Ethiopia. This study aimed to achieve several objectives: First, to assess the prevalence of workplace violence among nurses in Northern Ethiopia. Second, to identify potential factors associated with such violence.

The findings offer valuable insights into the prevalence, frequency, and nature of workplace violence (WPV) experienced by nurses. In this regard, this study can serve as a resource for policymakers in healthcare planning, particularly in developing violence prevention policies and protocols. Furthermore, the study will provide baseline data for future related research.

## Methods and materials

### Study setting

The study was conducted in all public hospitals located in Mekelle city, the capital of the Tigray Regional State in Northern Ethiopia. Mekelle city is situated ~780 km north of Addis Ababa, the Ethiopian capital. Mekelle is administratively divided into seven sub-cities, and the city boasts nine government health centers, four public hospitals (one primary and two general), one referral hospital. The study was conducted from December 20, 2023 to January 30, 2024. During the study period, these hospitals had a workforce of 1,214 nurses (12).

### Study design

A sequential explanatory mixed method study design was employed to conduct the study.

### Population

Nurses working in public hospitals in Mekelle city, Northern Ethiopia were included in the study. These nurses were actively involved in providing clinical care across various departments and specialties within these healthcare facilities. They have varying levels of experience, with some having just started their careers while others possess several years of professional practice.

### Eligibility criteria

Nurses who had been actively employed for at least 1 year in public hospitals in Mekelle were included in the study. However, nurses who were on maternity leave, sick leave, or annual leave during the study period were excluded from the study.

### Sample size determination

A single-population proportion formula was used to calculate the required sample size for this study. The prevalence of WPV (56%), established in a previous study conducted in northeast Ethiopia, served as the basis for the calculation. A 95% confidence level and a 5% margin of error were assumed (13). By adding 10% of the samples to compensate for the non-response rate, the minimum required sample size was four hundred sixteen (416).

For the qualitative study, participant recruitment continued until information saturation was reached. While the initial aim was to recruit 12 participants to ensure diverse perspectives, but 9 participants were ultimately interviewed for the IDIs and KIIs. This decision was based on the concept of inductive thematic saturation. In this approach, we analyze the collected data to see if there is new emerging codes or themes (14).

### Sampling technique

Computer-generated simple random sampling technique and critical case purposive sampling technique were used to select participants for the quantitative and qualitative parts, respectively.

In the qualitative part, six nurses were selected to participate in face-to-face interviews. These participants had all experienced workplace violence within the past 12 months. Three additional nurses were invited based on their position within the hospital.

### Data collection method and tools

Data for the quantitative approach were collected using self-administered questionnaire, while face to face in-depth interviews and key informant interview (KII) were used for the qualitative part.

The quantitative data was collected using a structured questionnaire developed in English and translated into Tigrigna, the local language. To ensure clarity and consistency, the questionnaire was translated by a team consisting of an English language speaker and a Tigrigna speaker. Finally, it was back-translated into English by a different person to verify its accuracy.

The tool was adapted from a data collection tool developed by the International Labor Office, International Council of Nurses, World Health Organization and Public Services International regarding WPV in the health sector. Modifications were made to fit the specific context of this study. The questionnaire covered respondents' socio-demographic characteristics, workplace features, and exposure to workplace violence (including perpetrator, location, and time).

For the qualitative part nine interviews held in hospitals, began with broad, open-ended questions followed by more precise and probing questions to gather information on the circumstances of the violence and perceived factors associated with the occurrence of violence. The principal investigator was conducting the in-depth interviews. The interviews were conducted in the local language (Tigrigna) and two trained research assistants were there for the tape recording and taking notes of all the in-depth interviews that were conducted. At first, the nurses who had faced WPV were invited. There were also announcements in departments that nurses who had experienced WPV could contact the research team to share their experiences. Among the nine participants, six were nursing officers, and the remaining three were key informants, including a vice chief nursing officer, a head nurse, and a nursing director who did not involve themselves in direct patient care. All the interviews were recorded by a voice recorder and saved in audio files in mp3 format, the minimum and maximum minute of the recorded interview was 25 and 34 min, respectively.

### Variables

#### Dependent variables

Workplace violence (Yes, No).

#### Independent variable

Sociodemographic characteristic (age, sex, marital status, educational level, religion).

Workplace characteristics (work experience, working unit, number of coworkers, shift work rotation, job satisfaction, work performance, working alone and training).

### Operational definitions

#### Workplace violence

Workplace violence Includes physical violence (such as kicking, hitting and pushing), psychological violence (such as verbal abuse, mobbing, or bullying), or sexual violence (such as sexual harassment, forced sex, or sexual acts/comments) (1). For the purpose of this study, workplace violence was considered present if participants experienced at least one type of work-related violence, 12 months prior to the study (11).

#### Work performance

Nine items on a 5-point Likert scale ranging from strongly disagree (1 point) to strongly agree (5 points) were used to measure work performance. Accordingly, good work performance was defined as a score that is greater than or equal to the mean value, and poor work performance was defined as a score that is less than the mean value (15).

#### Job satisfaction

Six items on a 5-point Likert scale ranging from strongly disagree (1 point) to strongly agree (5 points) were used to measure job satisfaction. Accordingly, satisfied was defined as a score that is greater than or equal to the mean value, and dissatisfied was defined as a score that is less than the mean value (15).

### Data management and analysis

Data were coded and entered into Epi Info version 7.2, and then exported to SPSS version 27 for statistical analysis. Both bivariate and multivariable logistic regression analyses were used to determine the association between the independent variables and dependent variable. In bivariate analysis variables having *p*-value < 0.2 were entered into a multivariable logistic regression to assess statistical association between the outcome variable and independent variables. In multivariable logistic regression, *p*-value < 0.05 with 95% confidence interval (CI) were considered statistically significant. The Hosmer-Lemeshow goodness-of-fit model coefficients tests procedure was used to test for model fitting. The assumption of logistic regression was checked before proceeding to regression.

For the qualitative part inductive thematic analysis approach with Coding reliability thematic analysis was employed. All in-depth interviews were recorded and transcribed using verbatim Transcribe software, and coded using ATLAS.ti 23. Researchers first coded the transcripts by identifying and marking important statements. These codes were then discussed by the team, and those codes with the same focus were combined into themes. This process resulted in the development of five key themes that shed light on the circumstances and perceived factors associated with the occurrence of violence among nurses. To protect the privacy of the participants, all names and public hospitals mentioned in this report have been replaced with codes.

### Data quality control

To ensure the quality of data, the questionnaire was prepared in English form, and then it was translated into Tigrigna (local language) and back-translated into English by experts to ensure its consistency.

However, to meet the objectives of the study, a pretest was done in 5% of the sample size in Wukro general hospital near the study area 5 days before the scheduled data collection day to improve the tool. The data collectors were trained for 1 day on principle, ethical considerations, and the meaning of the questions included in the questionnaire to standardize their collecting technique and strict supervision of the data collector was done by the supervisor and principal investigator. Each completed questionnaire was checked for errors, completeness and consistency immediately after collecting the data.

The qualitative study guaranteed the trustworthiness of the data, to ensure the credibility of the study, nurses of diverse genders, ages, and work experiences were recruited. These nurses had firsthand encounters with WPV in various settings. In-depth interviews were chosen as the data collection method to further strengthen the study's credibility. For authenticity, selected interviews along with their codes and categories were shared with two additional nurses, who confirmed that the codes accurately reflected real-life experiences. Additionally, detailed presentations of the findings and quotations were provided to enhance authenticity. Confirmability, was addressed by sending the results to the participants for their confirmation. They agreed that the findings accurately represented their experiences. Finally, the dependability of the research process, was ensured through open discussions within the research team regarding data analysis. Additionally, a researcher with expertise in qualitative research reviewed and approved the analysis.

## Result

Of the 416 nurses sampled from four government hospitals, 411 participated, yielding a response rate of 98.8%. In the qualitative study, the refusal rate was 0%.

### Sociodemographic characteristics of the respondents

Of the total respondents, 307 (74.7%) were females. One hundred and eighty seven (45.5%) aged 20–30 years. Three hundred and seventy three (90.8%) were Orthodox, and 250 (60.8%) were married. Three hundred and thirty eight (82.2%) held BSc degrees, and 177 (43.1%) had 6–10 years of work experience (Table 1).

**Table 1:** Sociodemographic characteristics of nurses working in Mekelle public hospitals, Mekelle, Tigray, Ethiopia 2023/24 (*n* = 416).

**Variables**	**Category**	**Frequency (n=416)**	**Percentage (%)**
Sex	Male	104	25.3
	Female	307	74.7
Age	20–30	187	45.5
	31–40	186	45.3
	>41	38	9.2
Religion	Orthodox	373	90.8
	Muslim	27	6.6
	Catholic	9	2.2
	Protestant	2	0.5
Marital status	Married	250	60.8
	Single	145	35.3
	Divorced	15	3.6
	Widowed	1	0.2
Level of education	Diploma	63	15.3
	Degree	338	82.2
	Masters	10	2.4
Work experience	< 1 year	1	0.2
	>1–5 year	111	27.0
	6–10 year	177	43.1
	11–15 year	72	17.5
	>15 year	50	12.2

### Workplace characteristics of the respondents

Out of the total respondents 384 (93.4%) worked in shifts, 238 (57.9%) worked with >15 workers in the same working area, 241 (58.6%) had good work performance and 232 (56.4%) respondents were dissatisfied with their job, finally 345 (83.9%) of the respondents claims that there is no reporting procedure for WPV in their working hospital (Table 2).

**Table 2:** Workplace characteristics of nurses working in Mekelle public hospitals Mekelle, Tigray, Ethiopia 2023/24 (*n* = 416).

**Variables**	**Category**	**Frequency (*n* = 416)**	**Percent (%)**
Working in shift	Yes	384	93.4
	No	27	6.6
Current working place	Medical	57	13.9
	Surgical	58	14.1
	Emergency	66	16.1
	Pediatrics	49	11.9
	ICU	40	9.7
	OPD	30	7.3
	OR	34	8.3
	Gyn Obs	11	2.7
	Psychiatry	6	1.5
	Orthopedics	16	3.9
	Clinics	23	5.6
	Other	21	5.1
No_ of staff	1–5	54	13.1
	6–10	50	12.2
	11–15	69	16.8
	>15	238	57.9
Reporting procedure	Available	66	16.1
	Not available	345	83.9
Job satisfaction	Dissatisfied	232	56.4
	Satisfied	179	43.6
Work performance	Poor	170	41.4
	Good	241	58.6
Trained on WPV	No	301	73.2
	Yes	110	26.8
Working alone	No	385	93.7
	Yes	26	6.3

### Prevalence of workplace violence

**Figure 1:**
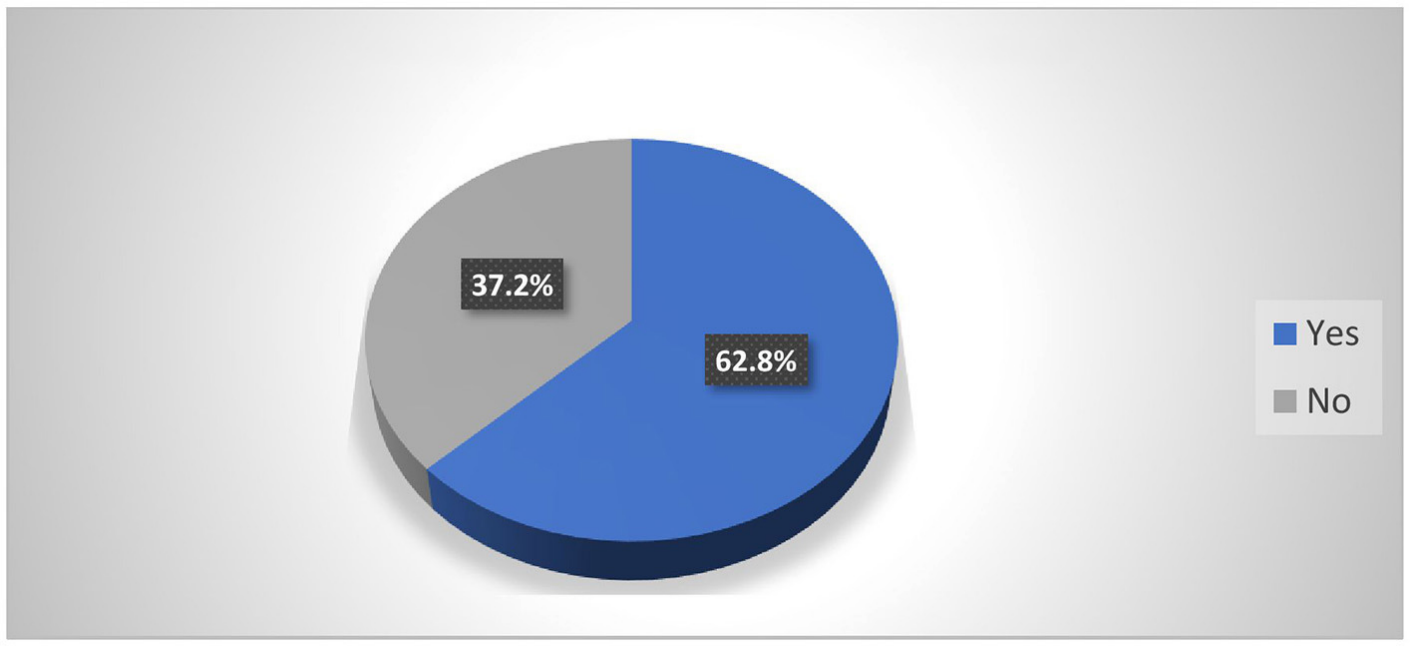
Prevalence of workplace violence among nurses working in Mekelle public hospitals Mekelle, Tigray, Ethiopia 2023/24 (*n* = 416).

**Figure 2:**
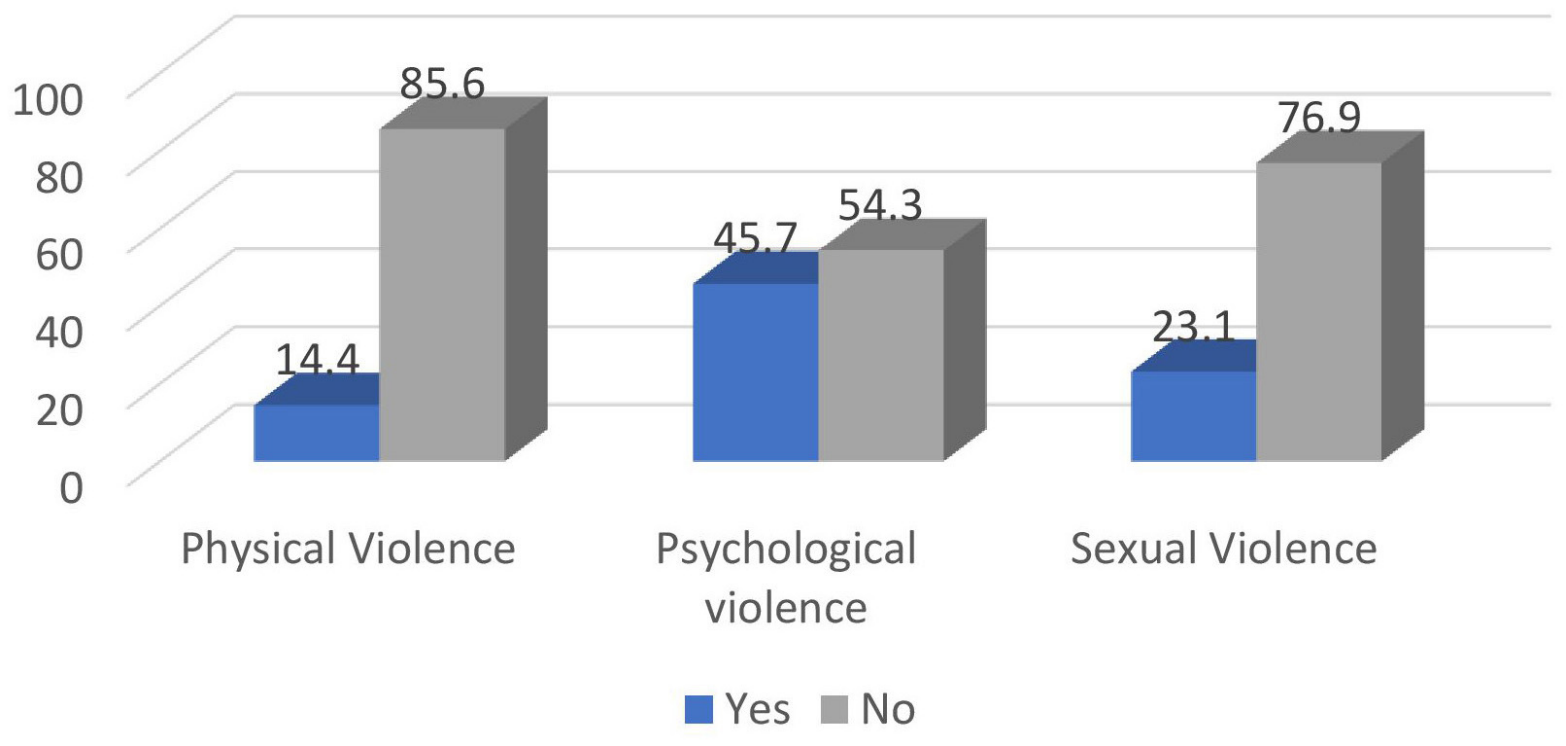
Prevalence of forms of workplace violence among nurses working in Mekelle public hospitals Mekelle, Tigray, Ethiopia 2023/24 (*n* = 416).

In the last 12 months, 258 (62.8%) of the participants had encountered at least one episode of WPV (verbal, physical, bullying, mobbing, and sexual) 95% CI [57.5, 76.3] (Figure 1). Out of the total participants, 188 (45.7%) reported psychological violence, 95 (23.1%) reported sexual violence, and 59 (14.4%) experienced physical violence (Figure 2).

#### Forms of workplace violence

**Figure 3:**
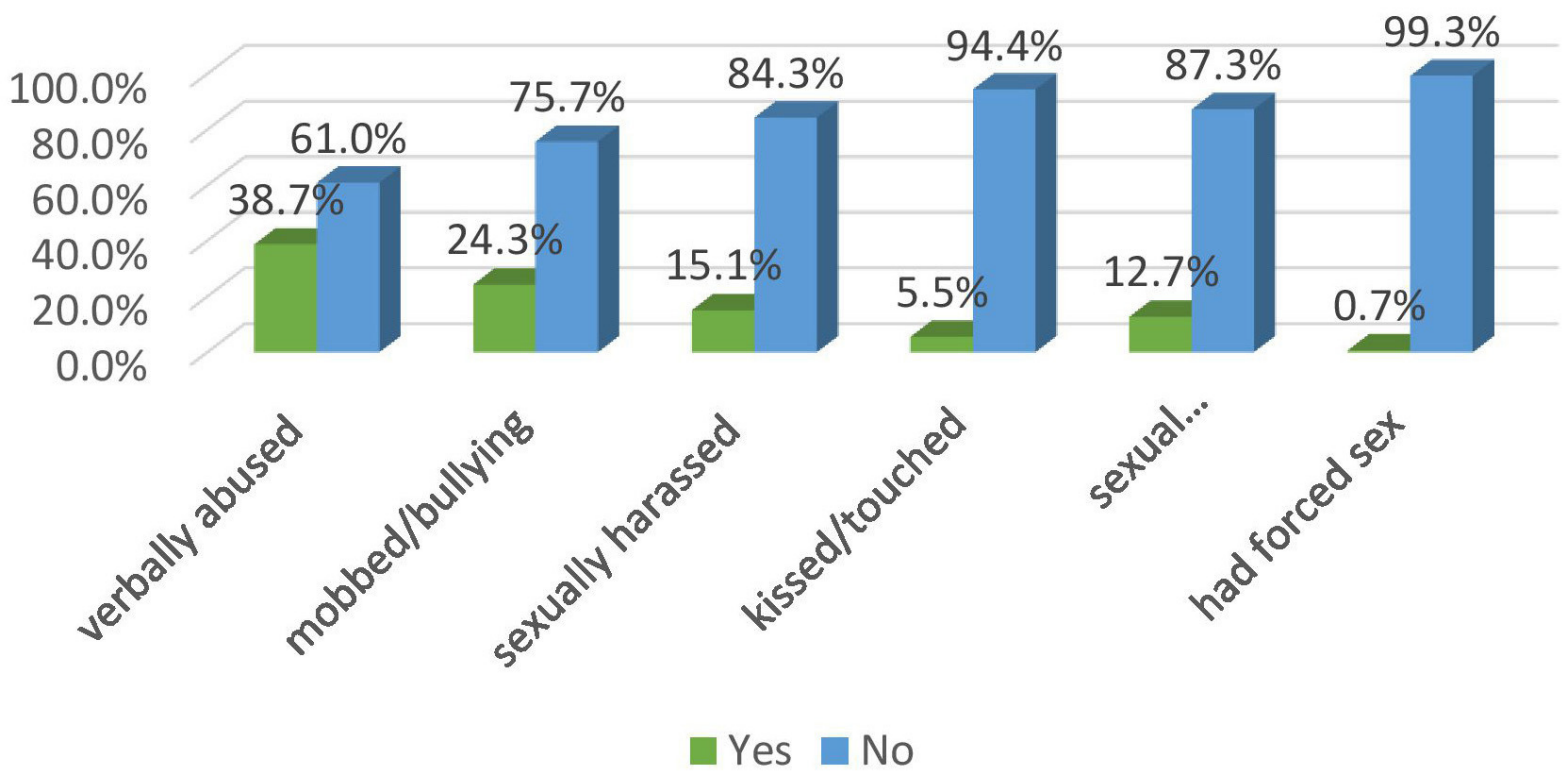
Prevalence of sub forms of workplace violence among nurses working in Mekelle public hospitals Mekelle, Tigray, Ethiopia 2023/24 (*n* = 416).

Among the total participants, 14.4% were physically attacked, 38.7% were verbally abused, 24.3% were mobbed or bullied, 15.1% were sexually harassed, 5.6% were kissed or touched sexually on their body, 5.6% faced unwanted sexual acts or comments and 0.7% of the respondents had forced sex (Figure 3).

#### Characteristics of the violence

From the total WPV encountered by the nurses, 132 (51.2%) were committed by relatives of patients, 61 (23.6%) and 56 (21.7%) happened in emergency and surgical wards, respectively. In addition to this 142 (55%) of the violence happened in the night shift and 73 (28.2%) of the violated participants encountered at least 2 episodes of violence in the last 12 months (Table 3).

**Table 3:** Characteristics of the violence among abused nurses working in Mekelle public hospitals Mekelle, Tigray, Ethiopia 2023/24 (*n* = 258).

**Characteristics**	**Category**	**Frequency (*n* = 258)**	**Percentage %**
Who attacked you	Patient	75	29.1
	Patients relative	132	51.2
	Staff	41	15.8
	Supervisor	10	3.9
Where did it happen	Emergency	61	23.6
	Surgical	56	21.7
	Medical	26	10.1
	Pediatrics	14	5.4
	Gyn obs	6	2.3
	OPD	21	8.1
	ICU	14	5.4
	OR	17	6.6
	Psychiatry	6	2.3
	Orthopedics	12	4.6
	Clinics	14	5.4
	Other	11	4.3
How many times	1 time	131	50.8
	2 times	73	28.2
	3 times	19	7.4
	>3 times	35	13.6
How did you respond	Took no action	101	39.1
	Told the person to stop	81	31.4
	Told family	13	5.0
	Tried to defend	21	8.1
	Told colleague	19	7.4
	Tried to pretend	17	6.6
	Pursued prosecution	6	2.3
When did it happen	Day shift	93	36.0
	Night shift	142	55.0
	Weekend	18	7.0
	Holyday	5	2.0

### Bivariate and multivariable results of workplace violence

In the multivariable analysis, current working place, marital status, work performance, number of staff, and working alone were found to be statistically significant factors with a *p*-value of < 0.05.

Married nurses were 42% less likely to encounter violence than single nurses [AOR 0.58 (0.34, 0.98)], besides this, nurse with good work performance were 41% less likely to experience violence than those with poor performance [AOR 0.59 (0.36, 0.96)].

Those nurses who worked with 1–5 staff members were three times more likely to experience violence in their workplace than those nurses worked with >15 nurses [AOR 3.4 (1.12, 10.4)]. Furthermore, those nurses worked in the emergency unit and surgical ward were 5 times and 10 times more likely, respectively, to encounter violence than those worked in the Gynecology and Obstetrics ward, AOR of [4.97 (1.1, 22.7)], [10.8 (2.14, 54.9)], respectively. Finally nurses who worked alone were two times more likely to be subjected to WPV than those nurses worked with more staff AOR of [2.34 (1.34, 4.1)] (Table 4).

**Table 4:** Bivariate and multivariable logistic regression result of factors associated with workplace violence in Mekelle public hospitals Mekelle, Tigai, Ethiopia 2023/24 (*n* = 416).

**Variables**	**Category**	**WPV**		**COR CI 95%**	**AOR CI 95%**	***P*-value**
		**Yes**	**No**			
No_ of staff in the same working area	1–5	41	13	1.66 (0.84, 3.27)^*^	3.4 (1.12, 10.4)^**^	0.03
	6–10	29	21	0.73 (0.39, 1.35)	1.1 (0.47, 2.68)	0.78
	11–15	32	37	0.46 (0.26, 0.78)^*^	0.6 (0.311, 1.2)	0.13
	>15	156	82	1	1	
Current working place**/**unit	Emergency	53	13	3.34 (0.9, 12.88)^*^	4.91 (1.1, 22.7)^**^	0.039
	Surgical	51	7	6.07 (1.46, 25.3)^*^	10.8 (2.1, 54.9)^**^	0.004
	ICU	15	25	0.51 (0.13, 1.93)	0.84 (0.18, 3.86)	0.82
	Medical	28	29	0.81 (0.22, 2.94)	1.21 (0.26, 5.34)	0.82
	OPD	21	9	1.94 (0.47, 8.05)	0.84 (0.16, 4.51)	0.84
	OR	23	11	1.74 (0.44, 6.97)	3.12 (0.64, 14.8)	0.16
	Orthopedics	10	6	1.42 (0.29, 6.62)	2.51 (0.43, 14.9)	0.31
	Other	9	12	0.63 (0.14, 2.71)	0.83 (0.16, 4.41)	0.83
	Clinics	19	10	1.62 (0.39, 6.51)	1.32 (0.26, 6.51)	0.75
	Pediatrics	23	26	0.19 (0.19, 2.74)	0.98 (0.22, 4.42)	0.98
	Gyn obes	6	5	1	1	
Work performance	Good	139	102	0.58 (0.39, 0.89)^*^	0.59 (0.4, 0.96)^**^	0.03
	Poor	119	51	1	1	
Working alone	No	202	99	1.97 (1.26, 3.06) ^*^	2.34 (1.34, 4.1) ^**^	0.003
	Yes	56	54	1	1	
Marital status	Married	153	113	0.52 (0.33, 0.79)^*^	0.58 (0.3, 0.99)^**^	0.046
	Unmarried	105	40	1	1	
Training on WPV	No	235	150	0.20 (0.06, 1.69) ^*^	0.08 (0.02, 1.3)	0.10
	Yes	23	3	1	1	
Age	>41	21	17	0.87 (0.43, 1.76)	1.44 (0.39, 5.31)	0.59
	20–30	128	59	1.53 (1.02, 2.34) ^*^	1.79 (0.97, 3.3)	0.06
	31–40	109	77	1	1	
Work experience	0–5	82	30	2.33 (1.16, 4.67)^*^	1.1 (0.28, 4.3)	0.91
	6–10	101	76	1.13 (0.61, 2.13)	0.71 (0.2, 2.468)	0.59
	11–15	48	24	1.72 (0.81, 3.57)^*^	1.64 (0.47, 5.72)	0.41
	>15	27	23	1	1	

^*^Statistically significant with p-value < 0.2, 1 = Reference group of the variable.

^**^Statistically significant with p-value < 0.05.

### Circumstances and perceived factors contributing to the occurrence of violence

Interviews were conducted with nine nurses regarding the circumstances of the violent incidents they experienced. From their descriptions, five common themes were identified which pointed out circumstances and perceived factors associated with the occurrence of violence; in presenting the results, quotes were used to reflect participants' voices.

### Theme 1: Violence behavior exists

The most commonly reported type of violence against nurses was verbal abuse, in addition mobbing and physical abuse were among the reported experience of WPV. Participants reported daily incidents of being shouted at or degraded by patients and their family members. Throwing objects and pushing nurses were common forms of physical violence. Insulting and humiliating were frequent experiences. Interviewees described that stress in the workplace, alcohol intoxication, lack of medications, dissatisfaction with the services provided, crowding, and long wait times were the most common triggers of violence. When patients are forced to wait, they are more likely to become agitated and engage in violent behavior. The narration of nurse “X”, at PH 1 describes violence that she has been faced in the previous 12 months…? in her words “*Verbal abuse and mobbing do occur, they are often rooted in current issues like lack of medication, and equipment access. But physical attacks are uncommon ultimately these verbal outbursts might stem from frustrations related to resource limitations*”.

The story of nurse “N”, a nurse at PH 2 describes violence that she has been faced in the previous 12 months…? in her words “*I have encountered violence firsthand. Although it hasn't involved sexual harassment or physical assault, I have personally experienced and witnessed a significant amount of verbal abuse and threats*. *Unfortunately, this issue persists even today*”.

### Theme 2: Perpetrators of violence

Family members or attendants were identified as the most common perpetrators of violence against nurses, also sometimes it is committed by patients. Many interview participants noted that violent events were more likely to take place at night, when lower staffing may result in longer wait times. Furthermore, many interviewees stated that hospital security staffing is also decreased during night shifts and perpetrators of violence against nurses may feel emboldened by the lack of security personnel and the most of the time it happens in emergency ward due to work overload and stress over there. Nurse “M” a female nurse at PH 3 said “*Violence perpetrated by relative is higher and mostly happen in night time*”, Nurse “A” a male head nurse at PH 2 said “*Most of the time, violence occurs in the emergency ward, where the workload is heavy due to the high number of patients. In contrast, other wards experience less violence. It mostly happens during night shifts, as people tend to become more irritable at night when they would normally be sleeping*”.

### Theme 3: Nurses' reaction to violence

The interviewed nurses confirmed during interviews that they didn't report their incidents, the main reason for this might be, that they feel ashamed, or it might be due to a lack of awareness concerning the report, even when they tried to report violent incidents, the hospitals management did not support them. Moreover, nurses confirmed that verbal violence was witnessed and also experienced frequently, but they did not report it and let it go, because they believed that reporting was useless and nothing would happen to address the issue, and most of them took no action against the event over the perpetrator.

Nurse “F” a male nurse at PH 4 said “*To whom to report this? and I'm concerned that reporting won't result in a response or solution*”.

The narration of nurse “Y” a nursing director at PH 1 describes the question “did you get reported any violence in the previous 12 months?” In his word “*I haven't get reported any violence yet. There could be a few reasons for this: some people might hesitate due to privacy concerns, feeling ashamed, or simply not being aware of the reporting process*”.

### Theme 4: Lack of management support

The hospitals didn't do anything to prevent work place violence there is no strong and cooperative guards, the weakness of hospital security let the free passage of the people. The hospital didn't respond immediately and didn't took any action if any incident happens. This issue is reflected in a series of quotation highlighted herein: nurse “Z” at PH 3 said “*The lack of effective leadership prevents action. The administration seems powerless to address our concerns*”. Another participant, nurse “B” at PH 4 said “*What's concerning is the lack of support for emergency department staff, considering the heightened risk of violence they face. This sensitive environment desperately needs dedicated security personnel, yet our requests have gone unanswered*”.

### Theme 5: Perceived factors triggering violence

Marital status, working unit, work performance, and working alone are not directly causative factors of violence among nurses but likely perceived contributors to violence.

Marital Status is unlikely to be a direct cause. However, work-life balance can be a stressor. Single parents or those with demanding home lives might experience higher stress, which could indirectly contribute to a shorter temper. This issue is reflected in a series of quotation, nurse “X” a nurse at PH 1 said “*Yes, there is a relation with marital status, it's possible that single nurses, often feeling more energetic, might react more directly to aggression. However, married individuals have other concerns, like their life status, which can teach them calm and effective ways to handle such situations*”.

Work Performance is another complex factor. Feeling pressure to perform exceptionally or fearing negative evaluations could heighten anxiety and contribute to a shorter fuse. However, equally likely is that nurses who experience violence might feel their performance suffers as a result. Nurse “G” a nursing head at PH 4 said “*Good work performance is definitely crucial because it directly strengthens the quality of patient care. Conversely, poor performance can lead to oversights, which, if ignored, put both patients and yourself at risk. Therefore, work performance plays a decisive role in patient safety and well-being*”.

Different units within a hospital can have vastly different work environments. An Emergency Room (ER) nurse might face more chaotic and potentially violent situations compared to a nurse in other units. The frequency and intensity of stressful interactions could play a role. “*Yes, emergency departments often experience high stress due to several factors. Patients arriving with critical conditions and trauma place a significant burden on the entire hospital system. Emergency staff can face challenging situations, including patient frustration and anger, inadequate security, and limited administrative support. These combined factors contribute to the heightened stress level in emergency wards*.” *According to* nurse “N” a nurse at PH 2.

Nurses working without immediate backup might feel more vulnerable to violence. Fear and a perceived lack of control can lead to aggressive responses in some situations. Nurse “F” a nurse at PH 4 said “*Stress can indeed be higher for people working alone in healthcare settings. Factors like potential patient dissatisfaction, difficulty with procedures like inserting cannulas without assistance, and potential negative reactions from patients due to these challenges can contribute to violence*”.

## Discussion

The prevalence of WPV among nurses working in public hospitals in Mekelle, Tigray, Ethiopia were consistent with the results of studies in Iran (67%) (8), Nigeria (66%) (16), Gambia (62.1%) (17), Egypt (59.7%) (18), northwest Ethiopia (63.5%) (19), northeast Ethiopia (58%) (13). This consistency across geographically diverse locations strengthens the argument that the prevalence of WPV among nurses in Mekelle might reflect a wider trend. It suggests that the issue may not be unique to Mekelle but could be a more general problem requiring attention. This suggests the need for national and institutional policies to prevent workplace violence (WPV), including zero-tolerance enforcement, effective reporting and support systems, and regular staff training on de-escalation and aggression management. Promoting a safety-focused organizational culture is also essential. Clinically, addressing WPV is critical for improving nurse retention, job satisfaction, and the overall quality of patient care, and should be integrated into routine clinical governance and occupational health practices.

On other hand the result of our study were lower compared to the study conducted in Kenya (77.8%) (5), Socio-cultural variations, discrepancies in healthcare systems, and potential under-reporting of incidents, could explain these disparities. This suggests ensuring accurate reporting and addressing systemic differences are essential steps toward creating safer work environments and improving healthcare worker wellbeing and patient care outcomes. Based on the qualitative study workplace violence is frequent but often underreported. Many nurses stated during interviews that hospital management provided little to no support when incidents were reported. Verbal abuse was commonly witnessed and experienced, yet frequently ignored, as nurses felt reporting was futile. Some female participants also shared that patient or their family members had attempted to touch them inappropriately, but they hesitated to report these incidents due to feelings of shame and humiliation.

The magnitude of WPV of this study was higher when compared to the study conducted in southern Ethiopia, Arba-Minch (43.1%) (10), This might be due to the differences that the people in recent times may enter to different socio-economic instabilities which may be taken as a pushing factor for violence that happened against nurses. This underscores the need for context-specific policy responses that consider broader social determinants of violence. The prevalence in this study was also higher compared to the study conducted in southern Ethiopia, Hawassa public health facilities (29.9%) (20). This might be due to the definition difference as the study in Hawassa used the last 6 months before the data collection to define workplace violence but this study used the last 12 months prior to the data collection to define workplace violence.

According to this study, married nurses were 42% less likely to have workplace violence than single nurses, in line with this, a study in southern Ethiopia found that individuals living alone were twice as likely to experience workplace violence compared to those living with a spouse (10). One possible explanation for this difference is that many nurses living alone is younger. Additionally, wearing a ring, especially a wedding band, may reduce the risk of certain types of harassment, such as sexual harassment. Based on the triangulated evidence derived from the qualitative findings of the present study, it was observed that nurses who are married appear to be less likely to experience workplace violence (WPV). This trend may be attributed to various social and psychological factors commonly associated with marital status, such as enhanced emotional support systems, greater stability in personal life, or increased maturity and coping strategies that can mitigate the risk or impact of violent encounters at work. Participants consistently highlighted that being in a committed relationship provided a sense of security and emotional resilience, which may contribute to their reduced vulnerability to WPV compared to their unmarried counterparts. Therefore, the qualitative result of the current study supports this.

Those nurses who worked with 1–5 staff members in the same working area were 3 times more likely to experience WPV than those nurses worked with >15 nurses in the same working area. Similar to this study, according to studies in northwest Ethiopia small number of staff (1–5) in the same working area were 2 times more likely to expose to WPV (2). This might be due to the fact that nurses with fewer colleagues in the same working area may face difficulty in dealing with and handling various job-related issues and additionally, may commit a mistake in giving nursing care. Based on the triangulated qualitative evidence from this study, nurses collaborating with more staff members showed improved handling and performance capabilities. Female nurses noted that working alone during certain shifts, being assigned to male wards, and the absence of male colleagues increased the risk of violence from patients and their relatives. This finding suggests that collaboration fosters positive outcomes in nursing practice. Therefore, the qualitative result of the current study supports this.

Furthermore, working in emergency unit and surgical ward was 5 times and 10 times more likely, respectively, to be associated with experiencing WPV than working in Gynecology and Obstetrics ward. This finding was similar to that of study conducted in Hawassa, which revealed that the odds of violence against nurses were nearly about 4 times higher among emergency department workers than those served in outpatient department. In addition to this, This finding was consistent with a study conducted in eastern Ethiopia, which revealed that nurses working in surgical wards (2x), psychiatric wards (3x), medical wards (5x), and emergency units (4x) were more likely to experience violence compared to those in specialized units (orthopedics, OR, recovery, burn, ANC, and others) (21). This might be due to potential workplace stress and high workload demands in the working department. According to the evidence triangulated from the qualitative result of the present study, working in the emergency unit poses a high risk of workplace violence due to the unit's sensitive nature. In addition, all the nurses believed that heavy workloads made it difficult to provide effective patient care and contributed to dissatisfaction among patients and their relatives. Therefore, the qualitative result of the current study supports this.

Our study indicates that nurse who have good work performance had 41% decreased odds of experiencing WPV compared to those who had poor work performance. This might be because those with good work performance might have the ability to manage things easily. According to the evidence triangulated from the qualitative result of the present study those with poor work performance may be more prone to making mistakes, often attributed to poor knowledge or skills While those with good work performance can navigate challenges more effectively. Some participants reported experiencing violence in response to their mistakes. Additionally, patients' perceptions of nurses as unskilled or lacking expertise in specialized care often triggered aggression. Interview data also suggested that patients sometimes believed nurses intended to harm them when they displayed insufficient knowledge or competence. Therefore, the qualitative result of the current study supports this.

## Limitations of the study

This study has some drawbacks. One is that nurses might not remember their experiences accurately. Another is that they may have been hesitant to share their full stories due to fear of consequences or negative judgment. This results in recall bias and social desirability bias due to some personal sensitive questions.

## Conclusion

This study found that the magnitude of workplace violence among nurses in Mekelle public hospitals was significantly high. Furthermore, factors such as marital status, number of staff in the same working area, work performance, working unit, and working alone, were identified as potential predictors of WPV among nurses. This emphasizes the need for targeted interventions to mitigate these risks and protect nurses in the workplace.

Workplace violence takes many forms and has various antecedents, including understaffing, shortages of drugs and supplies, lack of security, and inadequate management attention.

We recommend that implementing a strong system for preventing and reporting workplace violence incidents, with the involvement of medical staff, is essential for the health sector. Hospitals, policymakers, and other stakeholders must prioritize establishing a well-organized system for reporting workplace violence. This system should be designed with specific attention to the needs of vulnerable nurses, particularly females, less experienced and young nurses in high-risk wards. Finally, researchers interested in this area should conduct a purely qualitative study with a broader scope.

## Data Availability

The raw data supporting the conclusions of this article will be made available by the authors, without undue reservation.
